# Precision Tests of a Quantum Hall Effect Device DC Equivalent Circuit Using Double-Series and Triple-Series Connections

**DOI:** 10.6028/jres.100.050

**Published:** 1995

**Authors:** A. Jeffery, R. E. Elmquist, M. E. Cage

**Affiliations:** National Institute of Standards and Technology, Gaithersburg, MD 20899-0001

**Keywords:** ac quantum Hall effect, cryogenic current comparator, dc quantum Hall effect, equivalent electrical circuit, quantized Hall resistance, two-dimensional electron gas

## Abstract

Precision tests verify the dc equivalent circuit used by Ricketts and Kemeny to describe a quantum Hall effect device in terms of electrical circuit elements. The tests employ the use of cryogenic current comparators and the double-series and triple-series connection techniques of Delahaye. Verification of the dc equivalent circuit in double-series and triple-series connections is a necessary step in developing the ac quantum Hall effect as an intrinsic standard of resistance.

## 1. Introduction

In the integer dc quantum Hall effect [1–3] the Hall resistance *R*_H_ of the the plateau of a fully-quantized, two-dimensional electron gas (2DEG) is *R*_H_(*i*) = *h*/(*e*^2^*i*), where *h* is the Planck constant, *e* is the elementary charge, and *i* is an integer. We assume that *R*_H_(*i*) has the value of the von Klitzing constant, 25 812.807 Ω/*i*. The current flow within the 2DEG is nearly dissipationless in the quantum Hall plateau regions of high-quality devices, and the longitudinal voltage drops, *V_x_*, and the longitudinal resistance drops, *R_x_*, along the sides of the sample are very small.

**Fig. 1:**
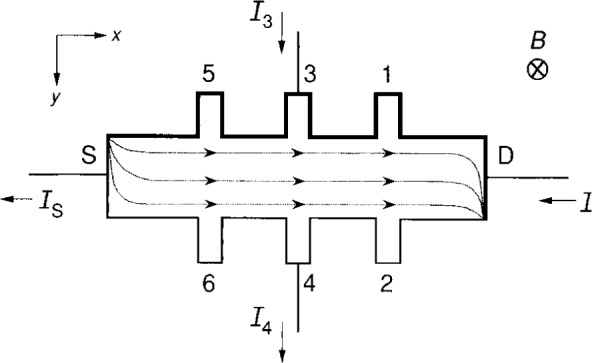
Top view of a quantum Hall device showing the direction of the current flow (positive carriers) in and out of the sample. The shaded curves indicate the electron flow pattern for a magnetic field pointing into the sample in the positive *z* direction. The bold lines indicate the positive potentials along the periphery of the sample.

It is important to remember that the conducting charges are *electrons*, and in the presence of a magnetic field, *B*, the sign and direction of the conducting charges determines the sign of the potentials around the periphery of the device. For the example shown in Fig. 1 the magnetic field is in the positive *z* direction, and the conducting electrons enter at the upper left-hand corner and exit at the lower right-hand corner [4–7], as indicated by the shaded curves. These corners remain the same on current reversal, but they interchange on magnetic field reversal. The potential probes 2, 4, and 6 are near the potential of the source S. Probes 1, 3, and 5 are near the potential of the drain D, and in this example have a positive potential relative to the source. The two positive potential sides of the device are indicated by thick lines. On current reversal, those two sides would have a negative potential relative to the source, and would be indicated by thin lines.

Electrons are moving from left to right in the example of Fig. 1, but electric circuit analyses traditionally assume currents composed of positive charges. Therefore the figure shows positively-charged currents *I*_D_ and *I*_S_ entering and leaving the device from right to left. If an external measurement system is connected to potential probes 3 and 4 to measure the quantum Hall voltage *V*_H_ ≡*V*_3_ –*V*_4_ = *R*_H_*I*_D_, then additional positively-charged currents *I*_3_ and *I*_4_ can enter and leave the device. These two currents are arbitrarily assumed to enter potential probe 3 and exit probe 4 in Fig. 1. *I*_3_ and *I*_4_ are small compared to *I*_D_ and *I*_S_.

## 2. The DC Equivalent Circuit

**Fig. 2:**
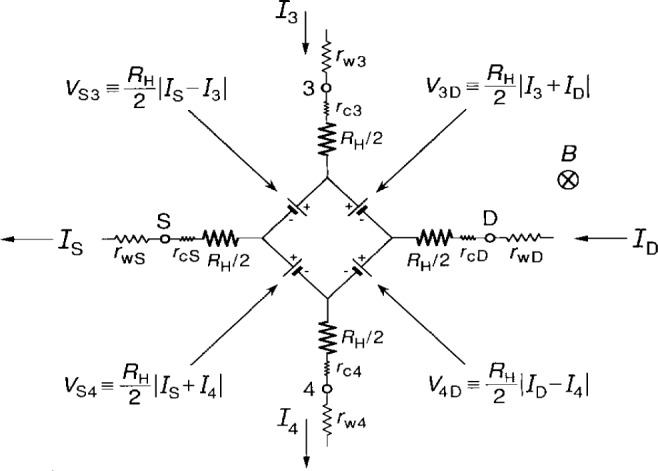
Equivalent circuit for the quantum Hall device of Fig. 1 with external leads to two potential contacts (3 and 4) and a source and drain (S and D). The orientations of the voltage generators are for the indicated current and magnetic field directions. The positions of the intrinsic resistances *R*_H_/2, contact resistances *r*_c_, lead resistances *r*_w_, and probe positions (S, D, 3, and 4), are also indicated.

Ricketts and Kemeny [8] have described the electrical behavior of a quantum Hall device in terms of an equivalent circuit. Figure 2 shows this equivalent circuit for the wiring configuration, current, and magnetic field directions of Fig. 1 when the longitudinal resistance *R_x_* is negligibly small. In ideal conditions, each arm of the circuit extends from the 2DEG to a source, drain, or potential contact, and has a large resistance *R*_H_/2 and a small contact resistance *r*_cA_ for each arm A, where A represents contacts, 3, 4, S, or D. There is also a wire resistance *r*_wA_ to each contact. The wire resistance is usually dominated by the sample probe leads, but it can also include wire-bonds to the sample header.

Between each pair of arms A and B there is a voltage generator *V*_AB_, where *V*_AB_ is defined as

$$V_{\text{AB}}\equiv \frac{R_{H}}{2}\left|I_{A}+I_{B}\right|.$$ (1)

The sign within the quantity |*I*_A_ + *I*_B_| is positive if both *I*_A_ and *I*_B_ enter or leave the device, and negative if one current enters and the other leaves. *V*_AB_ is zero if both *I*_A_ and *I*_B_ are zero.

**Fig. 3:**
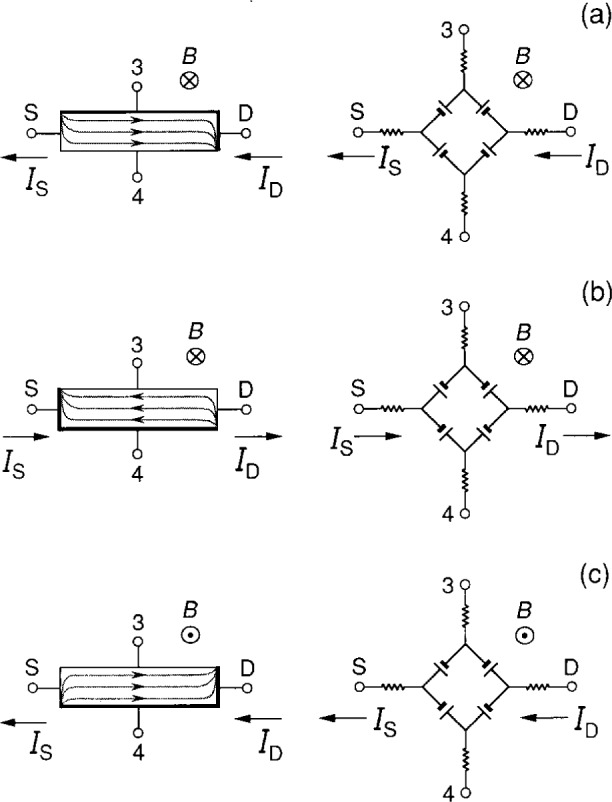
Diagrams of quantum Hall devices and the corresponding equivalent circuits to show how different magnetic field directions and different current flow directions affect the orientation of the voltage generators. (a) Positive current flow from D to S and a magnetic field in the positive *z* direction. (b) Current reversed and magnetic field unchanged. (c) Current unchanged from first case and magnetic field reversed. For simplicity, the contact resistances are not shown.

The currents *I*_A_ are assumed to be for positively-charged carriers, but the actual current is due to electrons traveling in the opposite direction, which in the presence of the magnetic field affects the signs of the potentials around the device periphery. Thus the orientations of the voltage generators are chosen in Fig. 2 to obtain the correct potentials. As shown in the simplified sketches of Fig. 3, the orientations (signs) of the voltage generators all reverse upon current reversal. They also all reverse on magnetic field reversal, but in that case the current directions remain the same.

Let us, for the moment, neglect the contact resistances *r*_cA_ and calculate the potentials *V*_3_, *V*_4_, and *V*_D_ at the device contacts 3, 4, and D in Fig. 2 relative to the source potential *V*_S_:

$$\begin{array}{c} V_{3}-V_{S}\approx I_{S}\frac{R_{H}}{2}V_{S3}+I_{3}\frac{R_{H}}{2}=\\ R_{H}I_{S}=R_{H}(I_{D}+I_{3}-I_{4}) \end{array}$$ (2a)

$$V_{4}-V_{S}\approx R_{H}I_{4}$$ (2b)

$$V_{D}-V_{S}\approx R_{H}(I_{S}-I_{3}).$$ (2c)

By subtracting Eq. (2b) from (2a), this leads to a correction to the quantum Hall voltage *V*_H_ ≡*R*_H_*I*_D_ of order *R*_H_*I*_3_. Even though *R*_H_ is large, the currents *I*_3_ and *I*_4_ can be made small enough in dc bridges to ensure that the correction to *V*_H_ is negligible. That is not necessarily the case, however, for bridges used to measure the ac quantized Hall resistance. Therefore something must be done in ac resistance measurements to ensure that *I*_3_ and *I*_4_ are indeed small enough. A solution, that has been found by Delahaye [9,10], involves double-series or triple-series connections to quantum Hall devices.

## 3. Double-Series Connections

**Fig. 4:**
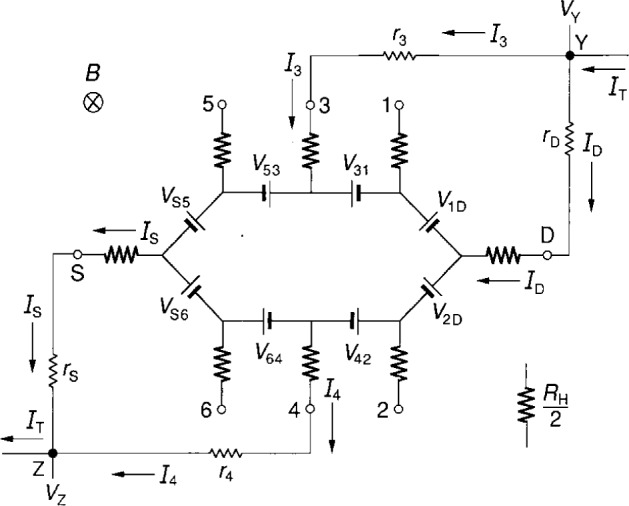
Double-series connection in an equivalent circuit representation of a quantum Hall device; *r*_S_, *r*_D_, *r*_3_, and *r*_4_ are defined in Eq. (3).

The total resistance in each arm, A, of the circuit can be split into the intrinsic resistance component *R*_H_/2 and the component *r*_A_, defined as

$$r_{A}\equiv r_{\text{cA}}+r_{\text{wA}}+r_{\text{test}}+r_{\text{CCC}},$$ (3)

where A represents S, D, 3 or 4, *r*_cA_ is the contact resistance, *r*_wA_ is the wire resistance, *r*_test_ is a resistor that can be added to test the circuit equations, and *r*_CCC_ is the resistance of a cryogenic current comparator that can be placed in arm A to measure the current *I*_A_. We display in Fig. 4 the equivalent circuit representation of two double-series connections to the quantum Hall device of Fig. 1, where *r*_A_ is defined by Eq. (3). A total current *I*_T_ enters point *Y*. It separates into currents *I*_3_, *I*_D_, *I*_4_, and *I*_S_, and then exits point *Z* as *I*_T_. By summing potentials around circuit loops one obtains the relations

$$I_{3}=\frac{r_{D}}{\left(R_{H}+r_{3}\right)}I_{D}=\frac{r_{D}}{\left(R_{H}+r_{D}+r_{3}\right)}I_{T}$$ (4)

and

$$I_{4}=\frac{r_{S}}{\left(R_{H}+r_{4}\right)}I_{S}=\frac{r_{S}}{\left(R_{H}+r_{S}+r_{4}\right)}I_{T}.$$ (5)

Therefore, *I*_3_ and *I*_4_ are small fractions of *I*_D_ and *I*_S_.

Four-terminal resistance measurements, *R*_Y,Z_, can be made between points Y and Z by using the two current leads and the potential leads *V*_Y_ and *V*_Z_:

$$R_{Y,Z}=\frac{V_{Y}-V_{Z}}{I_{T}},$$ (6)

or

$$R_{Y,Z}=R_{H}\left[1+\frac{r_{D}r_{3}}{R_{H}(R_{H}+r_{D}+r_{3})}+\frac{r_{S}r_{4}}{R_{H}(R_{H}+r_{S}+r_{4})}\right].$$ (7)

*R*_Y,Z_ differs from *R*_H_ by the two correction terms in Eq. (7), which we will label Δ*R*_H_(theory)/*R*_H_.

## 4. Triple-Series Connections

**Fig. 5:**
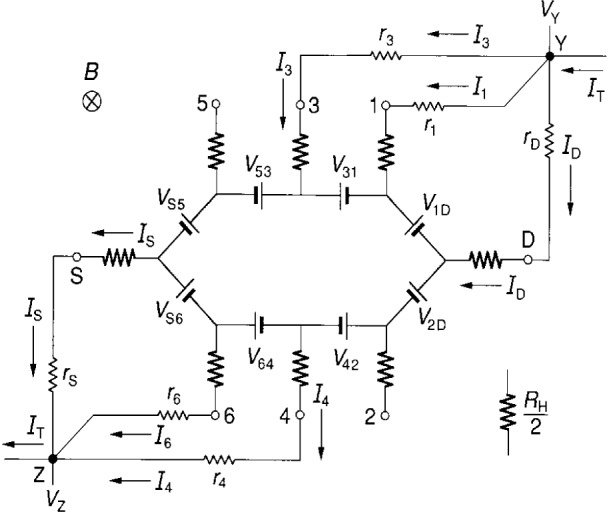
Triple-series connection in an equivalent circuit representation of a quantum Hall device.

Figure 5 shows the equivalent circuit representation of two triple-series connections to a single device. The equations are

$$I_{3}=\frac{r_{D}r_{1}}{\left[(R_{H}+r_{D}+r_{1})(R_{H}+r_{3})+r_{D}r_{1}\right]}I_{T},$$ (8)

$$I_{1}=\frac{r_{D}}{\left(R_{H}+r_{D}+r_{1}\right)}(I_{T}-I_{3}),$$ (9)

$$I_{4}=\frac{r_{S}r_{6}}{\left[(R_{H}+r_{S}+r_{6})(R_{H}+r_{4})+r_{S}r_{6}\right]}I_{T},$$ (10)

$$I_{6}=\frac{r_{S}}{\left(R_{H}+r_{S}+r_{6}\right)}(I_{T}-I_{4}),$$ (11)

and

$$\begin{array}{l} R_{Y,Z}=R_{H}\{1+\frac{r_{D}r_{1}r_{3}}{R_{H}[(R_{H}+r_{D}+r_{1})(R_{H}+r_{3})+r_{D}r_{1}}\\ \;+\frac{r_{S}r_{6}r_{4}}{R_{H}[(R_{H}+r_{S}+r_{6})(R_{H}+r_{4})+r_{S}]}\}. \end{array}$$ (12)

*R*_Y,Z_ differs from *R*_H_ by the two correction terms in Eq. (12), which we again label Δ*R*_H_(theory)/*R*_H_. Precision tests of these double-series and triple-series equations are described in Secs. 6 and 7.

## 5. Samples and Circuit Resistances

Two standards-quality samples were used. One sample, designated as GaAs(8), is a GaAs/Al*_x_*Ga_1–_*_x_*As heterostructure grown by molecular beam epitaxy at AT&T Bell Laboratories,^1^ with *x* = 0.29 being the fraction of aluminum atoms replacing gallium atoms in the crystal. It has a zero magnetic field mobility of about 100 000 cm^2^/(V·s) at 4.2 K, and a carrier density of 5.7×10^11^ cm^−2^. It has a length of 4.6 mm and a width of 0.4 mm, and the two outer Hall potential probe pairs are displaced from the central pair by 1 mm. Gold wires of diameter 25 µm were soldered onto alloyed indium dots to make electrical contact to the 2DEG.

The other sample is a BIPM/EUROMET GaAs/Al*_x_*Ga_1–_*_x_*As heterostructure grown by metalorganic vapor-phase epitaxy at the Laboratories d’Electronic Philips, France [11]. It is designated as E7C, has a Si_3_N_4_ protective coating, a zero magnetic field mobility of about 270 000 cm^2^/(V·s) at 4.2 K, a carrier density of 4.9×10^11^ cm^−2^, and an aluminum fraction of *x* = 0.29. It has a length of 2.2 mm and a width of 0.4 mm, and the two outer Hall potential probe pairs are displaced from the central pair by 0.5 mm. Gold wires of diameter 25 µm were wire-bonded onto enlarged Au/Cr contact pads evaporated onto the preexisting AuGe/Ni pads [12]. Both samples were mounted on 12-pin, T0-8 headers.

The samples were cooled in a He-3 refrigerator insert to a temperature of 0.3 K. The sample probe uses twisted-pairs of PTFE-covered copper thermocouple wire, whose diameters are only 76 µm to minimize heat loss. The resistances of these wires are about 10.0 Ω at room temperature and about 5.4 Ω when the sample is cold. There are two wires in the sample probe which are soldered together at the source connection of the T0-8 header, and two wires soldered together for the drain contact. The wire resistances *r*_wS_ and *r*_wD_ were determined by using one-half of the series resistances of the two source and the two drain wires, respectively. The wire resistances of the potential leads were then estimated by using the average values of *r*_wS_ and *r*_wD_ because all the wire resistances were equal to within 0.03 Ω. The wire resistances changed by as much as 0.2 Ω with liquid helium level, so it was important to monitor these resistances.

The contact resistances *r*_cA_ were determined from three-terminal resistance measurements. They were negligibly small for GaAs(8), and averaged about (0.17±0.10) Ω for the E7C sample. (Here, and throughout this paper, all quoted uncertainties are one standard deviation estimates.) The experiments were all done on the *i* = 2 plateau, so *R*_H_ = 12 906.403 5 Ω. The series resistance value of the 32-turn winding of a cryogenic current comparator, used in Sec. 6 to measure currents in selected leads of the sample probe, varied between 1.645 Ω and 1.650 Ω.

## 6. Current Measurements

Measurements were made of the current in selected potential leads of the GaAs(8) sample to determine if these currents agreed with the values predicted by Eqs. (4), (5), and (8) to (11) in Secs. 3 and 4. Both double-series and triple-series connections were used. The magnetic field direction was opposite to that for the equivalent circuits of Figs. 4 and 5, so the connections were on opposite sides of the sample. Both positive and negative currents were used for the applied current *I*_T_ in order to eliminate the effects of thermally-induced voltages. The experimental current in the potential lead was found using a 32-turn cryogenic current comparator (CCC) winding coupled to a dc-SQUID by measuring the SQUID flux-locked-loop output voltage. The sensitivity of this measurement is (38.5±0.2) nA/V.

### 6.1 Double-Series Connections

**Fig. 6:**
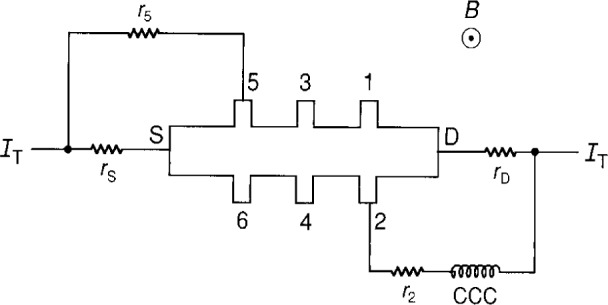
Double-series connection of a quantum Hall sample, and the position of a cryogenic current comparator (CCC) used to measure current in potential lead 2.

Results of the current measurements in potential leads are summarized in Table 1 for the double-series configuration shown in Fig. 6 and *I*_T_ = (39.914±0.001) µA. The agreement between measured and calculated values of the current are within the experimental uncertainty. No significant differences in currents measured (< 0.005 %) were observed for series-connections using different potential probe positions. Similar results were obtained if a double-series connection was made on only the side of the sample where the potential lead current was being measured.

Resistances with nominal values of 10 Ω and 20 Ω, and actual values of (9.989±0.002) Ω and (20.008±0.002) Ω, were added to the potential lead or the drain lead to observe their effect. Resistance added to the potential lead had little effect on the current. For example, when 20 Ω was added to potential lead 2, the measured current *I*_2_ decreased by only a factor of 5. When 10 Ω resistance was added to the drain lead, however, *I*_2_ increased by a factor of 2.8, and by a factor of 4.7 if 20 Ω was added.

The measurements are sensitive to changes in lead resistance (which depends on the liquid helium level), especially in the source or drain leads. An increase of 0.1 Ω in the drain lead resistance increases the calculated current by 1.8 % of the current.

We also obtained similar results when leads 1 and 5 were used in place of leads 5 and S of Fig. 6, and leads 6 and 2 in place of 2 and D. Therefore it does not matter what contacts are used as the source and drain as long as their contact resistances are comparable.

### 6.2 Triple-Series Connections

**Fig. 7:**
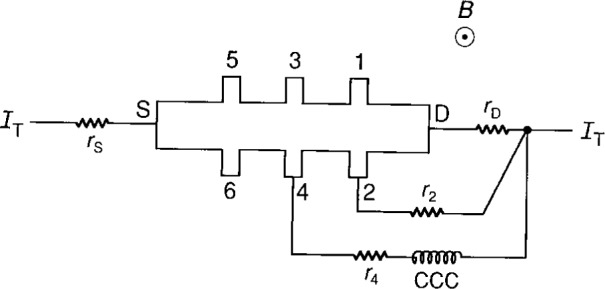
Triple-series connection of a quantum Hall sample, and the position of a CCC used to measure current in potential lead 4.

Current measurements were made using the triple-series connection to the drain side of the sample, as shown in Fig. 7. The results are summarized in Table 2 for the current in potential lead 4 and *I*_T_ = (39.914±0.001) µA. This example illustrates the extent to which the triple-series connection reduces the current in the potential leads. In fact, the current in lead 4 was so small (6×10^−12^ A) with no added resistance in the circuit that it was close to the detection limit of the measurement and could not be accurately compared with the theory, even when a 64-turn CCC winding was used to increase the sensitivity.

A better test of agreement between the measured and theoretical current values was obtained when large resistances (250 Ω and 500 Ω) were added to the drain lead or to potential lead 2. The large added resistances increased the currents in lead 4 by about two orders of magnitude, which was still about two orders of magnitude smaller than the double-series case. As a result, these current measurements do not agree as well with the calculated values as for the double-series connections. As predicted by Eq. (8), there is very little difference in the values of *I*_4_ whether resistance is added to the drain lead or to lead 2.

## 7. Resistance Measurements

A cryogenic current comparator measurement system, which compares the value of a *i* = 2 quantized Hall resistor to a 100 Ω resistor, was used for resistance measurements of sample E7C. Double-series and triple-series connections were employed, and resistances were again added to the drain lead to observe their effect on the quantized Hall resistance. The applied current was *I*_T_ = (30.702±0.001) µA, and the magnetic field direction was the same as in Figs. 4 and 5.

### 7.1 Double-Series Connections

**Fig. 8:**
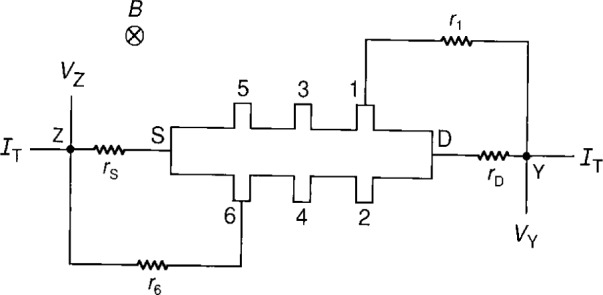
Double-series connection used for the resistance measurements of a quantum Hall sample. Measurements were made between points Y and Z.

The configuration for the double-series connection is shown in Fig. 8, and the results are in Table 3. Four-terminal double-series resistance measurements, *R*_Y,Z_, between points Y and Z are compared with regular quantum Hall measurements, *R*_H_, for potential probes 3 and 4 to obtain a relative experimental difference, Δ*R*_H_(exp)/*R*_H_, between double-series and regular quantum Hall measurements. The relative change from the regular quantized Hall resistance when using the double-series connection was 3.30×10^−7^ without any added resistance, 9.39×10^−7^ when a (20.008±0.002) Ω resistor was added to the drain, and 1.463×10^−6^ when a (37.045±0.002) Ω resistor was added.

The calculated relative differences Δ*R*_H_(theory)/*R*_H_ were obtained from Eq. (7). The relative differences

$$\delta R_{H}/R_{H}=\Delta R_{H}(\exp)/R_{H}-\Delta R_{H}(\text{theory})/R_{H}$$ (13)

between the experimental and calculated values for Δ*R*_H_/*R*_H_ in Table 3 range from 1.9×10^−8^ to 3.1×10^−8^, which is approximately four to six times the 5×10^−9^ relative experimental uncertainty of the 20 min duration CCC measurements. A likely explanation for these large values of δ*R*_H_/*R*_H_ is that the lead resistances, which were measured earlier in the day, increased significantly from those used in the calculations. For example, an increase in the lead resistances of 0.1 Ω, due to a decreasing liquid helium level, changes the predicted double-series-connected quantum Hall resistance values such that the three values of δ*R*_H_/*R*_H_ become 6×10^−9^, −3×10^−9^, and −3×10^−9^, respectively. To obtain reliable double-series measurements, the lead resistances need to be monitored frequently.

### 7.2 Triple-Series Connections

**Fig. 9:**
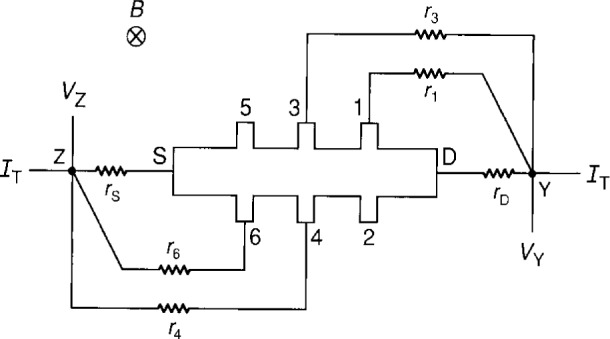
Triple-series connection used for the resistance measurements of a quantum Hall sample. Measurements were made between points Y and Z.

The triple-series connection is shown in Fig. 9, and the results of the measurements in Table 4. Four-terminal triple-series resistance measurements, *R*_Y,Z_, between points Y and Z are compared with regular quantum Hall measurements, *R*_H_, for potential probes 3 and 4 to obtain an experimental relative difference, Δ*R*_H_(exp)/*R*_H_, between triple-series and regular quantum Hall measurements. The calculated differences Δ*R*_H_(theory)/*R*_H_ were obtained from Eq. (12).

Δ*R*_H_(exp)/*R*_H_ and Δ*R*_H_(theory)/*R*_H_ are very small for triple-series connections. A 50 kΩ resistor had to be added in order to observe a significant relative change (1.24×10^−6^) from the actual quantized Hall resistance *R*_H_. Experimental and theoretical values are in excellent agreement, and differ only by 4×10^−9^.

## 8. Conclusions

The equations in Secs. 3 and 4, which are based on an equivalent circuit model of the quantum Hall device, give very good predictions for the current in the potential leads of the device, and for the change in the quantized Hall resistance when double-series and triple-series connections are employed. The double and triple-series connections have proved to be an effective means of reducing the current in the potential leads of a quantum Hall device, which is required in precision ac quantized Hall resistance measurements [10,13–15].

The triple-series connection is especially effective in reducing the current in the potential leads, even when very large resistances are added. Based on this, triple-series connections would be the better choice for use in high precision ac measurements.

The double-series and triple-series connections in this work were made outside of the liquid helium dewar. An alternative would be to make the series connections directly between the sample contacts. This would eliminate the effects of changes in the lead resistances due to variations in the liquid helium level and would reduce the number of leads required in the sample probe. It would, however, restrict one’s ability to make quantized Hall resistance measurements with reversed magnetic field, and to measure the longitudinal resistance.

It must be taken into consideration that the equivalent circuit model and the measurements are dc. It is not known for certain if this model will apply to high-accuracy ac measurements because no capacitive and inductive components are included in the model.

Four-terminal-pair connections [16] are used in high-precision ac measurements to ensure insensitivity to variations in series impedances and shunt admittances of the leads to the standard. A concern is that the resistance standard be composed of *R*_H_, and not a combination of *R*_H_ and other resistances, capacitances, and inductors. In a four-terminal-pair definition, the resistance standard is composed of the quantized Hall resistance and two of the coaxial cables used in the four-terminal-pair connections [16]. The impedances and admittances of the current and potential coaxial leads in the sample probe would therefore be part of the quantized Hall resistance standard [17] and would have to be accounted for, whether or not the double-series or triple-series connections were made at the sample contacts or outside of the dewar. Otherwise the resistance standard would not have the intrinsic value *R*_H_.

## Figures and Tables

**Table 1:** Experimental and calculated values of currents in the potential lead of a double-series connection with and without added test resistances. Theoretical current values are found from Eq. (4), but with probe 2 rather than probe 3, and the magnetic field reversed

Test resistance *r*_test_ (Ω)	Position of test resistance	Measured current *I*_2_ (nA)	Relative change in *I*_2_ from zero test resistance case (%)	Theoretical current *I*_2_ (nA)	Theoretical current ratio *I*_2_/*I*_T_ (%)	Theoretical current/measured current
0		16.77	0.0	16.90	0.042	1.01±0.01
20	Lead 2	16.74	−0.2	16.87	0.042	1.01±0.01
10	Drain lead	47.67	184.2	47.73	0.120	1.00±0.01
20	Drain lead	78.43	408.0	78.59	0.197	1.00±0.01

**Table 2:** Experimental and calculated values of currents in potential lead 4 of a triple-series connection with and without added test resistances. Theoretical current values are found from Eq. (8), but with probes 2 and 4 rather than probes 1 and 3, and the magnetic field reversed

Test resistance *r*_test_ (Ω)	Position of test resistance	Measured current *I*_4_ (nA)	Theoretical current *I*_4_(nA)	Theoretical current ratio *I*_4_/*I*_T_(10^−6^)	Theoretical current/measured current^a^
0		0.006	0.007	0.18	
250	Drain lead	0.32	0.33	8.24	1.03±0.01
500	Drain lead	0.62	0.64	16.00	1.02±0.01
250	Lead 2	0.32	0.33	8.16	1.02±0.01
500	Lead 2	0.63	0.63	15.84	1.01±0.01

a The value for the theoretical/experimental current ratio with no added resistance is 1.187, which is in much poorer agreement than observed with the double-series connection because the current in potential lead 4 for the case with no added resistance is four orders of magnitude smaller than the value obtained for the double-series connection—and is therefore close to the experimental detection noise. By adding large test resistances, the current was increased so that it was only two orders of magnitude less than the current measured for the double-series connection, and better theoretical/experimental ratios were obtained.

**Table 3:** Experimental and calculated values of the relative change in quantized Hall resistance, Δ*R*_H_/*R*_H_, from the usual quantum Hall measurement between potential probes 3 and 4 for a measurement between points Y and Z with a double-series connection. (See Sec.7.1 and Eq. (13) for the definitions of Δ*R*_H_(exp)/*R*_H_, Δ*R*_H_(theory)/*R*_H_ and δ*R*_H_/*R*_H_.)

Test resistance *r*_test_ (Ω)	Position of test resistance	Experimental result Δ*R*_H_(exp)/*R*_H_ (10^−6^)	Theoretical result Δ*R*_H_(theory)/*R*_H_(10^−6^)	Difference in results δ*R*_H_/*R*_H_ (10^−6^)
0		0.330	0.312	0.019
20	Drain lead	0.935	0.918	0.018
37	Drain lead	1.463	1.432	0.031

**Table 4:** Experimental and calculated values of the relative change in quantized Hall resistance, Δ*R*_H_/*R*_H_, from the usual quantum Hall measurement between potential probes 3 and 4 for a measurement between points Y and Z with a triple-series connection. (See Sec.7.1 and Eq. (13) for the definitions of Δ*R*_H_(exp)/*R*_H_, Δ*R*_H_(theory)/*R*_H_ and δ*R*_H_/*R*_H_)

Test resistance *r*_test_ (Ω)	Position of test resistance	Experimental result Δ*R*_H_(exp)/*R*_H_ (10^−6^)	Theoretical result Δ*R*_H_(theory)/*R*_H_ (10^−6^)	Difference in results δ*R*_H_/*R*_H_ (10^−6^)
37	Drain lead	−0.003	0.001	−0.004
50000	Drain lead	0.124	0.124	0.000

## Footnotes

1 Certain commercial equipment, instruments, or materials are identified in this paper to foster understanding. Such identification does not imply recommendation or endorsement by the National Institute of Standards and Technology, nor does it imply that the materials or equipment identified are necessarily the best available for the purpose.
